# PSMA PET/CT findings in high‐risk biochemical recurrence after local treatment of prostate cancer

**DOI:** 10.1002/bco2.70028

**Published:** 2025-05-12

**Authors:** Nicole Handa, Richard Bennett, Eric V. Li, Austin Ho, Mitchell M. Huang, Sai Kumar, Clayton Neill, Ridwan Alam, Hiten D. Patel, Edward M. Schaeffer, Ashley E. Ross

**Affiliations:** ^1^ Northwestern University, Feinberg School of Medicine Chicago IL USA; ^2^ Indiana University School of Medicine Indianapolis IN USA; ^3^ Surgery Service, Jesse Brown VA Medical Center Chicago IL USA

**Keywords:** biochemical recurrence, diagnostics, oligometastatic disease, prostate cancer, PSMA PET/CT

## Abstract

**Objectives:**

To describe PSMA PET/CT characteristics of patients with high‐risk BCR.

**Subjects/patients and methods:**

This was a retrospective analysis of patients with high‐risk BCR prostate cancer (PSA ≥ 2 ng/ml above nadir after radiation therapy [RT] or ≥1 ng/ml after radical prostatectomy [RP] +/− RT) who underwent PET/CT from July 2021–March 2023. Patients with prior cytotoxic chemotherapy, androgen deprivation therapy (ADT) initiated >3 months prior to PET/CT or positive conventional imaging within 3 months of PET/CT were excluded. Neoadjuvant/adjuvant ADT completed ≥9 months prior was allowed. Logistic regression, Pearson's Chi‐squared, Wilcoxon rank sum and Fisher's exact tests were used for analysis.

**Results:**

A total of 113 of 145 (77%) included patients in the analysis had ≥1 lesion on PSMA PET/CT. There was no difference in PSMA PET/CT positivity based on age, race, Gleason Grade at initial biopsy or PSA. Overall, 29 (20%) patients had lesions in the prostate/prostate bed only, 31 (21%) had lesions consistent with N1M0 disease and 53 (37%) had lesions consistent with M1 disease. For M1 patients, 21/53 (40%) had oligometastatic disease (1–3 lesions), and 32/53 (60%) had a higher burden (>3 lesions). Local recurrence was more common with RT and nodal recurrence with RP, with no difference in distant metastasis by initial treatment.

**Conclusion:**

Nearly 80% of patients with high‐risk BCR after local treatment for prostate cancer with RP and/or RT will have positive findings on PSMA PET/CT. In addition to intensified systemic therapy, up to 55% of the patients may have benefitted from salvage local therapy, nodal pelvic radiation or metastasis‐directed therapies for oligometastatic disease.

## 1 INTRODUCTION

Prostate cancer is the second most common type of cancer.^1^ While the long‐term oncologic outcomes are very favourable with treatment, up to one‐half of patients develop biochemical recurrence (BCR) at 5 years.^2^, ^3^ In the setting of BCR after presumptively complete local therapy, patients historically were managed with observation, intermittent androgen deprivation (ADT) or continuous ADT. As data developed in the metastatic setting demonstrating the benefit of intensification of therapy with androgen signalling inhibition, alternative treatment strategies were investigated for the management of BCR.^4^, ^5^, ^6^, ^7^ The EMBARK trial (NCT02319837) showed a benefit in PSA progression and metastasis‐free survival with enzalutamide plus leuprolide combination therapy and enzalutamide monotherapy compared to leuprolide alone among patients with high‐risk BCR.^8^ EMBARK allowed for a therapeutic “break” for patients with a pronounced response to therapy. Men receiving enzalutamide and ADT both were more likely to achieve a complete disease response and had the longest times off therapy. Despite this, the vast majority required re‐initiation of systemic therapy.

PSMA PET/CT is more accurate than conventional imaging and has been adopted by major guideline committees around the world as an option for the detection of disease in the setting of BCR.^9^, ^10^, ^11^, ^12^ Notably, the EMBARK trial utilized conventional imaging for enrolment, which could falter in the identification of low‐volume local‐regional or metastatic disease. Men with local‐regional disease may be eligible for local salvage therapies which can spare the morbidity of systemic therapies, and those with regional disease could benefit from the addition of radiation to their systemic therapy regimens.^13^, ^14^, ^15^ Further, data suggests that oligometastatic disease detected by PSMA PET/CT could be treated with metastasis‐directed therapy (i.e. stereotactic radiation), resulting in disease remission in many cases.^16^, ^17^, ^18^ Here, we sought to characterize sites of disease recurrence on PSMA PET/CT for patients with high‐risk BCR.

## 2 SUBJECTS/PATIENTS AND METHODS

### 2.1 Patient selection

This was a retrospective analysis of patients who underwent RP and/or RT for the treatment of prostate cancer and developed high‐risk BCR at our institution between July 2021 and March 2023. High‐risk BCR was defined as PSA ≥ 2 ng/ml above nadir after RT or ≥1 ng/ml after RP +/− adjuvant RT, similar to the EMBARK trial.^8^ PSA doubling time (PSADT) and testosterone were not evaluated as part of the inclusion criteria, as the information was not available for all patients. After being diagnosed with high‐risk BCR, patients underwent ^68^Ga‐PSMA‐11 or ^18^F‐DCFPyL PET/CT. Patients were excluded if they had undergone prior cytotoxic chemotherapy, androgen deprivation therapy (ADT) initiated >3 months prior to PSMA PET except for neoadjuvant/adjuvant ADT completed ≥9 months prior, or had positive conventional imaging within 3 months of PSMA PET/CT. This study was approved by the Northwestern University Institutional Review Board (STU00211144) and performed in accordance with the Declaration of Helsinki.

### 2.2 Outcomes

Patterns of PSMA PET/CT avidity were characterized based on location. Local recurrence was defined as PSMA PET/CT avidity in the prostate or prostate bed. Nodal recurrence was defined as PSMA PET/CT avidity in regional lymph nodes only. Metastatic recurrence was stratified into patients that had oligometastatic recurrence with ≤3 lesions and those that had >3 sites of recurrence based on PSMA PET/CT avidity. M1 recurrence was further stratified into M1a disease with cancer that has spread to distant lymph nodes outside of the pelvis, M1b disease with spread to the bone and M1c disease meaning cancer has spread to other organs.

### 2.3 Statistical analysis

Continuous variables were summarized as median and interquartile range and compared between the groups using the Wilcoxon rank sum test. Categorical variables were summarized as frequencies and percentages and were compared with Fisher's exact test and the chi‐squared test, as appropriate. Logistic regression analysis was performed to determine the association between the outcome of a positive PSMA PET/CT and the initial treatment strategy. Multinominal logistic regression was performed to determine associations between disease location on PSMA PET/CT (localized, nodal and distant) and the initial treatment strategy. A subset analysis was completed for patients that did have data available for PSADT, focusing on those with a PSADT <9 months since that was part of the inclusion criteria for the EMBARK trial.^8^ Statistical significance was set at P < 0.05. The analysis was completed using R version 4.2.0.

## 3 RESULTS

**FIGURE 1:**
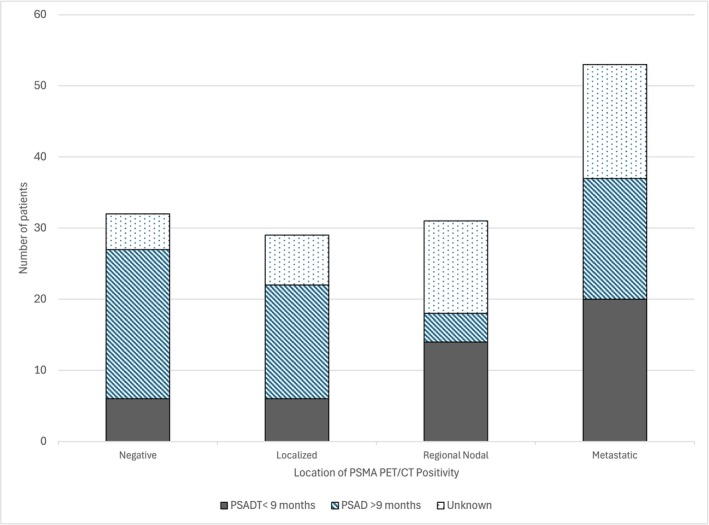
PSMA PET/CT positivity by location stratified by prostate‐specific antigen doubling time (PSADT).

We observed 145 patients who demonstrated high‐risk BCR after initial treatment for prostate cancer. There were 113 (77%) patients who had at least one PET‐avid lesion identified on PSMA PET/CT, of whom 29 had local recurrence, 31 had nodal recurrence and 53 had distant metastatic recurrence (Figure 1). Based on areas of PSMA PET/CT avidity in our cohort, 20/53 patients had M1a disease, 31/53 patients had M1b disease, 2/53 patients with M1c disease. There were 32 patients (23%) with no evidence of recurrence on PSMA PET/CT. When comparing patients with a positive PSMA PET/CT and those with a negative PSMA PET/CT, we found no significant difference in age, race, grade group on initial biopsy or PSA at the time of PSMA PET/CT (Table 1).

**TABLE 1:** Patient characteristics prior to PSMA PET/CT by imaging findings.

Characteristic	Negative (N = 32)^1^	Positive (N = 113)^1^	P‐value^2^
**Age**	69.50 (66.00, 77.50)	73.00 (66.00, 78.00)	0.6
**Race**			0.6
Asian	1 (3.1%)	2 (1.8%)	
Black	3 (9.4%)	16 (14%)	
White	24 (75%)	77 (66%)	
Unknown	4 (12.5%)	20 (18.1%)	
**Charlson Comorbidity Index**	5.50 (5.00, 9.50)	6.00 (5.00, 9.00)	0.5
**PSA at PSMA PET/CT**	2.32 (1.41, 4.36)	3.81 (1.72, 6.15)	0.045
**logPSA at PSMA PET/CT**	0.84 (0.34, 1.47)	1.34 (0.54, 1.82)	0.045
**PSA Categorical at PSMA PET/CT**			0.12
< 4	23 (72%)	58 (51%)	
4–10	6 (19%)	35 (31%)	
≥ 10	3 (9.4%)	20 (18%)	
**Initial Treatment**			0.023
RP	14 (44%)	63 (56%)	
RT	7 (22%)	35 (31%)	
RP + RT	11 (34%)	15 (13%)	
**Gleason Grade at biopsy**			>0.9
1	0 (0%)	2 (5.7%)	
2	2 (33%)	7 (20%)	
3	1 (17%)	7 (20%)	
4	1 (17%)	8 (23%)	
5	2 (33%)	11 (31%)	
Unknown	26	78	
**Gleason Grade at RP**			0.2
1	5 (24%)	5 (6.8%)	
2	4 (19%)	14 (19%)	
3	7 (33%)	22 (30%)	
4	1 (4.8%)	9 (12%)	
5	4 (19%)	23 (32%)	
Unknown/Underwent RT monotherapy	11	40	
**Tumour Stage at RP**			0.042
pT2	11 (55%)	18 (25%)	
pT3a	5 (25%)	29 (41%)	
pT3b	4 (20%)	24 (34%)	
pTX/Underwent RT monotherapy	12	42	
**Node Stage at RP**			0.064
pN0	18 (90%)	57 (81%)	
pN1	0 (0%)	11 (16%)	
pNX/Underwent RT monotherapy	14	45	

^1^ n (%); Median (Q1, Q3).

^2^ Pearson's Chi‐squared test; Wilcoxon rank sum test; Fisher's exact test.

There were 77 (53%) patients who underwent RP, 42 (29%) who underwent RT and 26 (18%) who underwent RP plus RT. The median PSA at time of PSMA PET/CT for patients who underwent RP, RT and RP plus RT was 2.70 ng/ml, 4.68 ng/ml and 2.05 ng/ml, respectively. PSMA PET/CT was positive in 63 patients (81.8%) who underwent RP, 35 patients (83.3%) who underwent RT and 15 patients (57.7%) who underwent RP plus RT (P = 0.023). On multivariable logistic regression controlling for PSA at the time of PSMA PET/CT, the use of combination RP and RT at the time of initial therapy was associated with a significantly lower risk of a positive PSMA PET/CT when compared to RP alone (OR 0.31, 95% CI 0.11–0.82, P = 0.02). There was no difference between the RP and RT monotherapy groups (OR 0.91, 95% CI 0.32–2.70, P = 0.90).

The distribution of disease recurrence differed based on initial treatment strategy. Multinominal logistic regression demonstrated that the odds of a localized recurrence were significantly higher in patients who received RT than in those who received RP (OR 4.50, 95% CI 1.31–15.42, P = 0.02) **(**Table 2 **)**. When compared to the RP group, patients who received RT monotherapy and RP plus RT were 92% (OR 0.08, 95% CI 0.01–0.69, P = 0.02) and 80% (OR 0.20, 95% CI 0.05–0.73, P = 0.02) less likely, respectively, to demonstrate a nodal recurrence. The proportion of distant metastatic disease did not differ significantly among the three treatment groups.

**TABLE 2:** Multinomial logistic regression to determine associations between the location of disease recurrence on PSMA PET/CT (local, nodal or distant) and initial treatment strategy.

Location of PSMA PET/CT avidity	Treatment	N	OR^1^	95% CI ^1^	P‐value
**Prostate/Prostate Bed (Local)**					<0.001 ^2^
	**RP**	77	‐	‐	
	**RT**	42	4.501	1.313, 15.42	0.017
	**RP + RT**	26	0.477	0.102, 2.235	0.3
**Nodal (Regional)**					<0.001 ^2^
	**RP**	77	‐	‐	
	**RT**	42	0.077	0.009, 0.690	0.022
	**RP + RT**	26	0.196	0.053, 0.730	0.015
**Distant Metastasis**					<0.001 ^2^
	**RP**	77	‐	‐	
	**RT**	42	1.104	0.370, 3.294	0.9
	**RP + RT**	26	0.351	0.115, 1.067	0.065

^1^ OR = Odds Ratio, CI = Confidence Interval.

^2^ Global p‐value for the categorical variable.

Within the subset of the 53 patients with distant metastatic lesions, 21 (40%) had 1–3 lesions and 32 (60%) had >3 lesions. There was no significant difference in number of lesions based on age, race, initial treatment type, pathologic stage at initial treatment or PSA at the time of PSMA PET/CT (Table 3).

**TABLE 3:** Characteristics of patients prior to PSMA PET/CT with distant metastases.

Characteristic	1–3 lesions (N = 21)^1^	>3 lesions (N = 32)^1^	P‐value^2^
**Initial Treatment**			0.7
RP	10 (48%)	19 (59%)	
RT	7 (33%)	9 (28%)	
RP + RT	4 (19%)	4 (13%)	
**Age**	74 (66, 78)	71 (65.5, 76.5)	0.5
**Race**			0.7
Asian	0 (0%)	0%	
Black	4 (19%)	4 (13%)	
White	14 (67%)	20 (63%)	
Unknown	3 (14%)	8 (24%)	
**Charlson Comorbidity Index**	9 (6, 10)	6.5 (5, 10.5)	0.5
**PSA at PSMA PET/CT**	4.8 (1.64, 5.80)	4.54 (2.65, 12.86)	0.2
**logPSA at PSMA PET/CT**	1.57 (0.49, 1.76)	1.51 (0.97, 2.55)	0.2
**PSA Categorical at PSMA PET/CT**			0.033
< 4	9 (43%)	14 (44%)	
4–10	11 (52%)	8 (25%)	
≥ 10	1 (4.8%)	10 (31%)	
**Gleason Grade at RP**			>0.9
1	1 (7.7%)	2 (8.7%)	
2	2 (15%)	3 (13%)	
3	4 (31%)	8 (35%)	
4	2 (15%)	4 (17%)	
5	4 (31%)	6 (26%)	
Unknown/Underwent RT monotherapy	8	9	
**Tumour Stage at RP**			0.076
pT2	0 (0%)	7 (32%)	
pT3a	6 (50%)	6 (27%)	
pT3b	6 (50%)	9 (41%)	
pTX/Underwent RT monotherapy	9	10	
**Node Stage at RP**			0.6
pN0	9 (75%)	19 (86%)	
pN1	2 (25%)	3 (14%)	
pNX/Underwent RT monotherapy	9	10	

^1^ n (%); Median (Q1, Q3).

^2^ Fisher's exact test; Wilcoxon rank sum test.

Within the overall study population, there were 104 patients with PSADT, of which 46 patients had a PSADT <9 months. In this subset of 46 patients, 6 (13.0%) had no lesions, 6 (13.0%) had local recurrence, 14 (30.4%) had nodal recurrence and 20 (43.5%) had metastatic disease on PSMA PET/CT (Figure 1).

## 4 DISCUSSION

Recent advances have shown that patients with high‐risk BCR can benefit from early administration of next‐generation systemic hormonal therapies such as enzalutamide. However, this treatment is not without side effects, and patients with localized and oligometastatic disease may have a chance at curative treatment. As such, we were interested in characterizing the distribution of sites of recurrence on PSMA PET/CT to determine if certain patients could be candidates for localized salvage therapy.^8^ Based loosely on the EMBARK inclusion criteria, this study showed that about 80% of patients with high‐risk BCR will have a positive lesion on PSMA PET/CT, with over half of patients demonstrating localized, regional nodal or oligometastatic disease. [8] In the subset of patients with PSADT <9 months the rates of PSMA PET/CT positivity were even higher, around 87%, similarly with over half of patients demonstrating localized, regional nodal or oligometastatic disease. For the patients with no visible disease on PSMA PET/CT or >3 metastatic lesions, there should be strong consideration of intensified systemic treatment with ADT + ARPI. Based on the EMBARK trial, it is reasonable to consider a defined treatment course with ongoing monitoring to limit the morbidity of treatment. For patients with localized or regional nodal recurrence, morbidity associated with systemic therapy may be avoided altogether or delayed with alternative therapies. Treatment options for these patients include salvage RP, RT, selective node dissection, ablative therapy or focal brachytherapy.^13^

Over one third of patients in this study had at least one site of distant metastatic recurrence. While the current standard is for these patients to be treated with intensified systemic therapy and ADT, the question remains whether there are alternative treatments that can delay disease progression with decreased risk of toxicity.^10^ In one small retrospective study of 20 patients with BCR after radical prostatectomy and 3 or fewer oligometastases on PSMA PET/CT treated with MDT, 74% of patients were able to have ADT postponed for 2 years.^19^ The POPSTAR, STOMP and ORIOLE trials have demonstrated that there may be some benefit over observation and suggest that metastasis‐directed therapy may delay time to ADT.^10^, ^16^, ^17^, ^18^, ^20^ Stereotactic ablative radiotherapy after RP or RT is an option for patients with regional or oligometastatic recurrence based on the current AUA/ASTRO/SUO guidelines with Grade C evidence.^10^ Further research is needed to evaluate long‐term oncologic outcomes and adverse events. To evaluate how many patients might be good candidates for localized metastasis‐directed treatment, the number of sites of metastatic disease were investigated. Overall, 14% of patients had oligometastatic disease with 1–3 metastatic lesions. The most common sites of metastatic PSMA PET/CT avidity were the bones and distant lymph nodes. These patients may be eligible for metastasis‐directed therapy to delay disease progression and increase time to systemic therapy.^19^

While patients that underwent RP or RT had similar rates of PSMA PET/CT positive lesions, patients that underwent both RP plus RT had lower rates of subsequent PSMA PET/CT positive lesions, suggesting a potential benefit to adjuvant or early salvage therapy, which is consistent with other studies.^21^, ^22^ In combination, RP plus RT may work to decrease any micro‐metastatic disease that is present at the time of initial treatment. However, this intensified adjuvant therapy is often reserved for those with very high‐risk features at the time of initial diagnosis, as combination RP with RT is associated with an increased risk of urinary incontinence, bladder neck contractures.^23^, ^24^, ^25^ On the other hand, salvage RT for patients with a rise in PSA after RT may be curative if initiated early. Despite the data supporting salvage RT, not all patients opt to proceed with salvage RT or may have comorbidities making them a poor candidate for pelvic RT. In our retrospective RP cohort, PSA levels rose to 1 ng/ml or higher, but it is important to note that these patients may have been cured of their prostate cancer if early salvage RT was initiated prior to a PSA level of 0.5 ng/ml and that salvage therapy should be offered.^26^, ^27^

As PSMA PET becomes more widely available and used routinely for the workup of BCR, it is important to consider how to harness the information it provides to improve patient outcomes.^28^, ^29^ One way is to identify sites of recurrence that are not necessarily visible on conventional imaging and consider metastasis‐directed treatment to those sites rather than starting systemic therapy. There is also a continued need to understand the role of PSMA PET/CT versus conventional imaging in the biochemical recurrence space given that many of the therapies were initially approved and efficacy was determined based on conventional imaging findings. Furthermore, future studies should continue to investigate long‐term oncologic outcomes for patients treated with metastasis‐directed therapies based on PSMA PET/CT findings.

There are limitations of this study. Given that it was a retrospective analysis at a single tertiary care centre, many of the patients were referred and had their initial workup and treatment elsewhere. Therefore, not all data was reliably available for these patients, and some variables, such as PSADT, could only be analysed for a subset of patients. Additionally, based on prior studies, it is widely accepted that there can be false positives and false negatives on PSMA PET/CT and performance varies by PSA level.^9^, ^30^ In this study, there was no histopathologic confirmation of the presence of cancer in the PSMA‐avid lesions. The cutoff PSA cutoff of >1 ng/ml for patients that had undergone RP is also relatively high, and many providers may consider starting therapy sooner. However, this threshold was selected based on the inclusion criteria of the EMBARK trial as a threshold for which there is evidence of benefit for intensification of systemic therapy and is reflected in current guidelines.^8^, ^11^ There is still no clear consensus on the absolute PSA value at which salvage therapies should be initiated. Similarly, further study is needed to determine optimal timing for systemic therapy initiation and treatment regimens.^13^ At the time of PSMA PET/CT, 22% of patients had >3 metastatic lesions using the PSA thresholds in this study, suggesting that earlier intervention would have been beneficial.

## 5 CONCLUSION

Overall, based on PSMA PET/CT findings in this study, up to 55% of patients could have delayed or avoided intensified systemic therapy and its potential side effects with alternative treatment options. 14% of patients with a PSA of >0.2 ng/ml over the nadir post‐radiation therapy or PSA > 1 ng/ml post‐radical prostatectomy with or without radiation had oligometastatic disease with 1–3 lesions on PSMA PET/CT making them potential candidates for metastasis‐directed therapies. Furthermore, 20% of patients had local recurrence and 21% had nodal recurrence making them candidates for salvage therapies. This adds to the growing body of literature examining PSMA PET/CT findings in the setting of biochemical recurrent prostate cancer and supports its use to inform treatment decisions.

## AUTHOR CONTRIBUTIONS

*Study concept and design:* Nicole Handa, Edward M. Schaeffer, and Ashley E. Ross. *Acquisition of data:* Richard Bennett, Eric V. Li, Austin Ho, and Clayton Neill. *Analysis and interpretation of data:* Nicole Handa, Richard Bennett, Eric V. Li, Mitchell M. Huang, Sai Kumar, Hiten D. Patel, and Ashley E. Ross. *Drafting of manuscript:* Nicole Handa. *Critical revision for important intellectual content:* Richard Bennett, Eric V. Li, Mitchell M. Huang, Ridwan Alam, Hiten D. Patel, Edward M. Schaeffer, and Ashley E. Ross. *Statistical analysis:* Sai Kumar. *Supervision:* Edward M. Schaeffer and Ashley E. Ross.

## CONFLICT OF INTEREST STATEMENT

HDP is supported by a Prostate Cancer Foundation Young Investigator Award (24YOUN22) and a Developmental Research Program grant from the SPORE in Prostate Cancer (P50 CA 180995). HDP served on a one‐time advisory board for Cleveland Diagnostics. EMS is a consultant for Pfizer and PinnacleCare Health Advisors. AER is a consultant for Astellas, Astra Zeneca, Bayer HealthCare Pharmaceuticals, BillionToOne, Janssen Biotech, Lantheus, Myovant, Novartis, Pfizer, and Veracyte. All other authors have no declarations of interests to report.
